# Composition and Nutritious Properties of Coffee

**Published:** 1846-08

**Authors:** 


*Composition and Nutritious Properties of Coffee.* By M. Payen.—M. Cadet de Vaux and M. C. de Gassicourt fancied they had discovered in coffee gallic acid, a resinous substance, albumen, an aromatic essence, and mucilage; these substances present, however, only doubtful analogies with the well-defined elements of coffee. Caffeine, discovered by Runge, and described by Robiquet, is a crystallisable and azotised body, easily sublimated into white brilliant crystals, and appears identical in nature with theine, since detected in tea-leaves. Robiquet, besides, described with precision the effects of torrefaction on coffee. The researches of Liebig appeared to show that coffee contained in reality but little or no nutritious matter; and M. Payen, on the contrary, endeavors to demonstrate that the infusion of coffee contains several azotised principles, in quantity equal at least to ten times the amount admitted by Liebig; and that saline and fatty substances, of a nutritious nature, may also be therein detected. According to M. Payne, the most remarkable of the immediate principles of coffee had not hitherto been discovered—a fact which is accounted for by the extreme facility with which that principle is altered by chemical operations. Its most interesting alteration consists in the production of a fine green colour, which betrayed the presence of the unknown substance to M. Payen, and led to its separation in the shape of a white crystalline matter, which imparts a deep green hue to five thousand times its weight of water or alcohol. M. Payen has by experiment ascertained that torrefaction, according to its degree, removes from coffee 15 to 25 per cent. in weight, and adds to its volume from 30 to 50 per cent. With regard to the nutritious properties of the infusion, M. Payen asserts that the infusion of 100 grammes of coffee in 1000 of water contains 20 grammes of nutritious matter, three times more than the same quantity of tea, prepared with 20 grammes to 1000 of water. If milk be added to it, the result will contain six times more solid matter and three times more nitrogen than broth.—*Ibid.*

